# Deep learning differentiates myeloproliferative neoplasms and predicts *JAK2* and *CALR* mutations from bone marrow smears

**DOI:** 10.1007/s00277-026-07257-w

**Published:** 2026-08-25

**Authors:** Jan-Niklas Eckardt, Chethan Babu V. Reddy, Holger Hauspurg, Sebastian Riechert, Tim Schmittmann, Ishan Srivastava, Susann Winter, Katja Sockel, Markus Badstübner, Martin Bornhäuser, Markus Tiemann, Jan Moritz Middeke

**Affiliations:** 1 Department of Internal Medicine I, University Hospital Carl Gustav Carus, TUD Dresden University of Technology, Fetscherstraße 74, Dresden, 01307 Germany; 2 Else Kröner Fresenius Center for Digital Health, TUD Dresden University of Technology, Dresden, Germany; 3 Mildred Scheel Early Career Center, National Center for Tumor Diseases (NCT/UCC) Dresden, Faculty of Medicine and University Hospital Carl Gustav Carus, TUD Dresden University of Technology, Dresden, Germany; 4 Cancilico GmbH, Dresden, Germany; 5 Institute for Hematopathology Hamburg, Hamburg, Germany; 6 German Cancer Consortium (DKTK), Partner Site Dresden, and German Cancer Research Center (DKFZ), Heidelberg, Germany; 7 National Center for Tumor Diseases Dresden (NCT), NCT//UCC Dresden, a partnership between DKFZ, Faculty of Medicine and University Hospital Carl Gustav Carus, TUD Dresden University of Technology, and Helmholtz-Zentrum Dresden-Rossendorf (HZDR), Dresden, Germany

**Keywords:** Myeloproliferative neoplasms, Polycythemia vera, Essential thrombocythemia, Myelofibrosis, Deep learning

## Abstract

Accurate differentiation of BCR::ABL1-negative myeloproliferative neoplasms (MPNs) based on bone marrow smear morphology remains challenging. While previous artificial intelligence (AI) approaches have primarily focused on bone marrow core biopsies, the diagnostic potential of routine bone marrow smears has remained largely unexplored. Using 460 digitized bone marrow smear whole-slide images, we developed an attention-based multi-instance learning model using features extracted from a self-supervised vision foundation model to classify polycythemia vera (PV), essential thrombocythemia (ET), and primary myelofibrosis (PMF) and to predict *JAK2* and *CALR* mutation status. Our model achieved a receiver-operating-characteristic area-under-the-curve (ROC-AUC) of 0.84 for MPN subtype classification and predicted *JAK2* and *CALR* mutation status in ET (ROC-AUC 0.73 and 0.75) and PMF (ROC-AUC 0.67 and 0.70) based on bone marrow smear images only. Our results provide a proof of-concept for AI-supported bone marrow cytomorphology as a modality complementary to core biopsies in MPN diagnostics and suggest that driver mutations leave traceable morphological signatures in smears. Multimodal integration of microscopic imaging with laboratory and clinical findings may further boost MPN differentiation performance of diagnostic AI models.

**Supplementary Information:**

The online version contains supplementary material available at https://doi.org/10.1007/s00277-026-07257-w.

To the Editor

Myeloproliferative Neoplasms (MPN) encompass a set of hematopoietic stem cell disorders that share genetic driver alterations impacting signaling pathways critical for hematopoiesis. [1] As a part of the BCR::ABL1-negative MPN complex, polycythemia vera (PV), essential thrombocythemia (ET) and primary myelofibrosis (PMF) show changes of laboratory values, constitutional symptoms, and altered bone marrow morphology upon initial diagnosis. The evaluation of the latter is crucial in the diagnostic workup and requires experienced hematopathologists to detect morphologies that warrant the diagnosis of either PV, ET, or PMF. The visual assessment is further underpinned by the detection of three key molecular alterations driving the disease: Mutations of *JAK2*, *CALR*, and/or *MPL*. [2, 3].

The rise of artificial intelligence (AI) and its current merger with the medical domain offer a unique set of possibilities for digital applications to improve the speed and accuracy of diagnosis and develop digital biomarkers for targeted therapies. [4] In this study, we developed an AI pipeline, distinguishing PV, ET, and PMF in bone marrow smears (BMS) and predicting mutation status of *JAK2* and *CALR*.

Whole slide images (WSI) from 460 BMS (174 ET, 110 PV, 176 PMF of which 91 were pre-fibrotic and 85 were overt fibrotic) were digitized using a Sysmex P1000 scanner at 40x magnification (0.25 μm/pixel). Tile extraction was performed using SlideFlow [5] with a tile size of 224 × 224 pixels. Blur-based filtering and Otsu thresholding were used to exclude regions with poor scanning focus and/or background areas. For feature extraction, we used Virchow [6], a self-supervised transformer-based vision encoder foundation model trained specifically on large-scale histopathology data, which leverages knowledge distillation and contrastive learning principles to learn semantically rich, domain-specific image features without requiring manual annotations. The resulting 2048-dimensional feature vectors were subsequently used for slide-level classification using a customized attention-based multi-instance-learning (MIL) model (Fig. 1). Binary cross-entropy with logits loss was used as the objective function, learning rate was governed by the Adam [7] optimizer, and hyperparameters were tuned with the Optuna [8] framework. Threefold internal cross-validation was used with strict patient-level splits. Performance was evaluated using macro-averaged receiver operating characteristic area under the curve (ROC-AUC), accuracy, and F1 score. ROC-AUC curves were generated for each cross-validation fold. All metrics are reported as mean ± standard deviation (std) across folds.

**Fig. 1:**
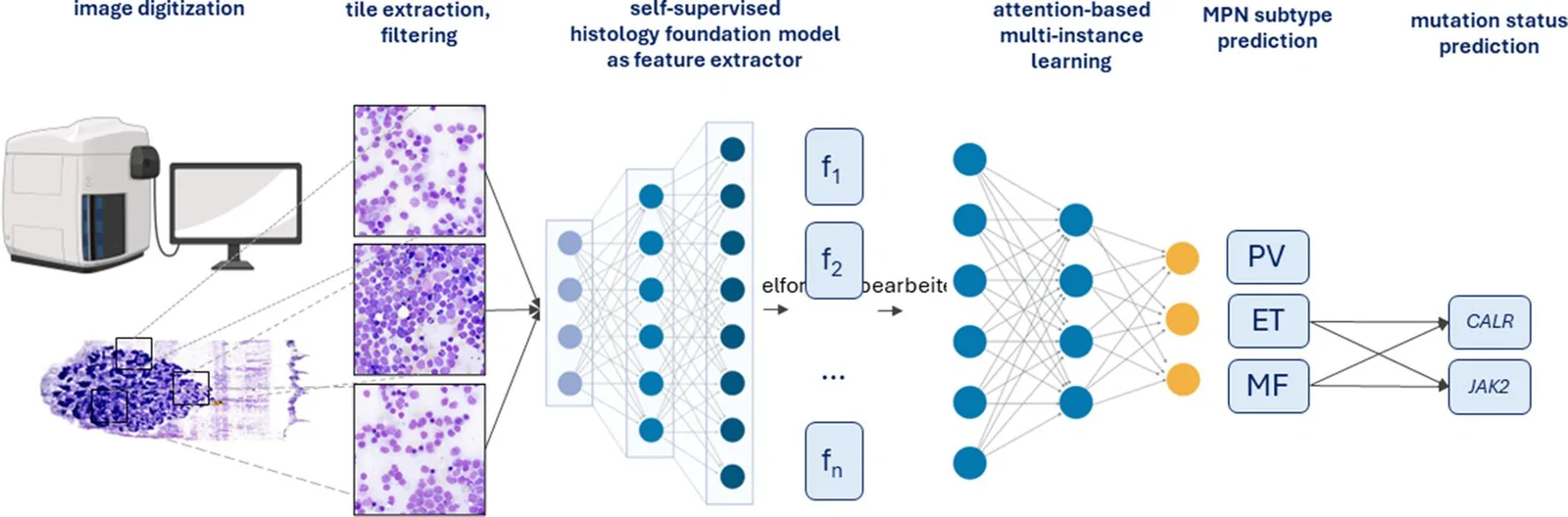
Workflow for Diagnostic Subtyping and Mutation Status Prediction.Bone marrow smears were digitized as whole slide images at 40x magnification. Extracted tiles were subsequently used as input to Virchow, a self-supervised histology foundation model to autonomously extract image feature vectors. These were then used as an input for an attention-based multi-instance learning (MIL) model that first differentiated between polycythemia vera (PV), essential thrombocythemia (ET), and primary myelofibrosis (PMF). In ET and PMF images, the MIL model was additionally trained to predict *JAK2* and *CALR* mutation status

Training and internal validation were conducted with a 2:1 split. For BCR::ABL1-negative MPN subtyping, classification performance showed a mean macro-averaged ROC-AUC of 0.84 (std ± 0.01), a mean macro-averaged F1 score of 0.68 (std ± 0.01), and a mean accuracy of 0.75 (std ± 0.02; Table S1, Fig. 2). PV was most reliably detected with a ROC-AUC of 0.92 (std ± 0.0), while ET and PMF yielded ROC-AUCs of 0.81 (std ± 0.01) and 0.79 (std ± 0.02), respectively. Given subtype specific differences in frequencies of *JAK2*, *CALR*, and *MPL* mutations showing either highly dysbalanced data (*JAK2* in PV) or very few training samples (*MPL* in all subtypes, *CALR* in PV), not all possible combinations could be tested. For *JAK2* prediction in ET, we obtained a macro-averaged ROC-AUC of 0.73 (std ± 0.01), a mean macro-averaged F1 score of 0.61 (std ± 0.02), and a mean accuracy of 0.66 (std ± 0.03; Table S2, Fig. 2). For *CALR* in ET, macro-averaged ROC-AUC was 0.75 (std ± 0.03), mean macro-averaged F1 score was 0.66 (std ± 0.04), and mean accuracy was 0.72 (std ± 0.05; Table S3, Fig. 2). For *JAK2* in PMF, macro-averaged ROC-AUC was 0.67 (std ± 0.03), mean macro-averaged F1 score was 0.64 (std ± 0.03), and mean accuracy was 0.66 (std ± 0.04; Table S4, Fig. 2). Lastly *CALR* prediction in PMF showed a macro-averaged ROC-AUC of 0.70 (std ± 0.02), a mean macro-averaged F1 score of 0.63 (std ± 0.03), and a mean accuracy of 0.66 (std ± 0.05; Table S5, Fig. 2).

**Fig. 2:**
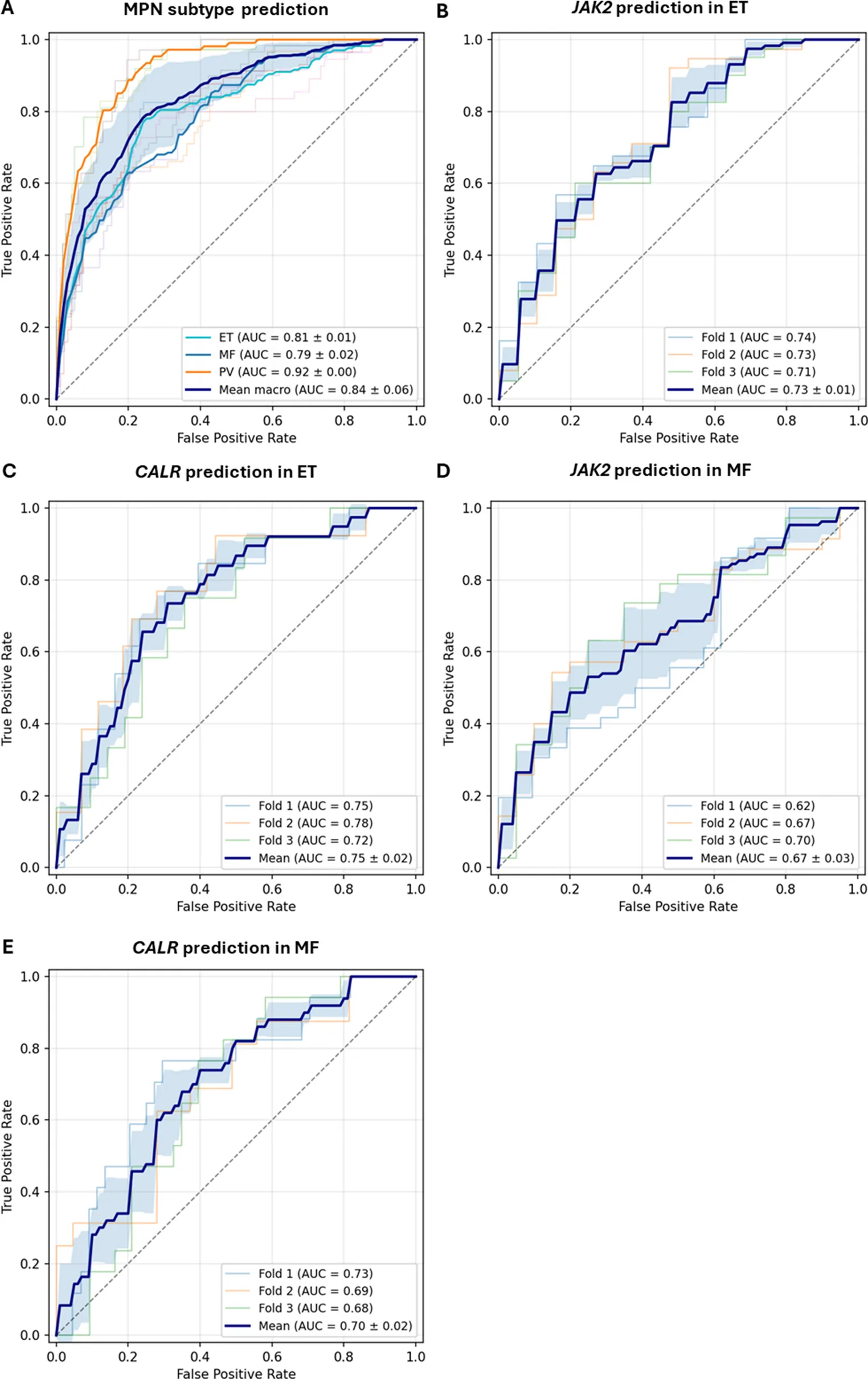
Model performance in predicting subtypes of myeloproliferative neoplasms and mutation status of *JAK2* and *CALR*. Internal three-fold cross-validation was performed for all classification tasks. Our model accurately subtyped myeloproliferative neoplasms (MPN) in bone marrow smears (**A**), showing the highest area under the curve (AUC) for the detection of polycythemia vera (PV), followed by essential thrombocythemia (ET) and primary myelofibrosis (PMF). Predicting the mutation status of *JAK2* and *CALR* in ET and PMF from bone marrow smears (**B**-**E**) showed moderate to good performance. Detailed metrics are provided in supplementary tables S1-5

AI-based computer vision can distinguish between PV, ET, and PMF based on BMS microscopy image data and predicts *JAK2* and *CALR* mutation status in ET and PMF with moderate accuracy. Regarding BCR::ABL1-negative MPN subtyping, our findings are in line with several studies differentiating PV, ET, and PMF, using either peripheral blood smears or, more frequently, bone marrow core biopsies. In peripheral blood smears, Kimura et al. [9] trained a model on complete blood counts, features extracted by an automated cell count system, as well as features extracted by a convolutional neural net, and reported sensitivity and specificity both over 0.9 for differentiating MPNs. Combining morphological features (e.g., myeloid-to-erythroid ratio and megakaryocyte morphology) from bone marrow core biopsies with clinical features, Yu et al. [10] trained random forest classifiers, reporting an AUROC of 0.96 and precision of 0.81 in differentiating BCR::ABL1-negative MPNs. Similarly, Wang et al. [11] combined BCR::ABL1-negative MPN bone marrow core biopsies and clinical features in a fusion model and compared model outputs with hematopathologists, reporting model diagnostic accuracy to be on par with senior hematopathologists. Sirinukunwattana et al. [12] used principal component analysis of megakaryocyte morphologies from bone marrow core biopsies, reporting high discriminative accuracy between MPNs and reactive specimens. In a subsequent study by the same group [13], megakaryocyte feature analysis paired with continuous indexing of fibrosis allowed discrimination between pre-fibrotic MF and ET. Krichevsky et al. [14] evaluated multiple models to predict *JAK2*,* CALR*,* TET2*, and *ASXL1* mutation status on BCR::ABL1-negative MPN bone marrow core biopsies, reporting high prediction accuracy with MIL-based models.

These findings are limited by their retrospective nature, and both prospective and third-party external validation are warranted before safe clinical implementation is possible. Unimodal models such as ours or from previously conducted studies should, however, only be considered proof-of-concept towards multimodal diagnostic models incorporating clinical findings, laboratory values and histology, thereby covering WHO/ICC [2, 3] diagnostic standards and, potentially, supporting diagnostic decision-making with cytomorphology and digital biomarkers. As could be expected from real-world experience, our model showed the highest diagnostic performance for identification of PV, while delineating ET and PMF was more difficult. A key challenge in hematopathology is differentiating between pre-fibrotic MF and ET which could not be appropriately addressed due to the small sample size in our study. Multimodal models trained on a larger sample size incorporating core biopsies, smears, laboratory values and clinical findings could help to differentiate between pre-fibrotic MF and ET. Further, we only delineated BCR::ABL1-negative MPNs among each other while reactive conditions and other hematological disorders may mimic morphologic bone marrow states found in MPNs. Future work will therefore encompass a broader set of disorders to increase real-world clinical applicability. Both our and Krichevsky’s [14] findings further yield the possibility of image-based genetic subtyping. While such digital biomarkers will likely not replace molecular testing, they may complement it by providing a fast initial screening in the diagnostic work-up, prompting the diagnostician to run the appropriate molecular tests.

In summary, we developed a MIL-based model to differentiate between BCR::ABL1-negative MPNs and predict *JAK2* and *CALR* mutation status in ET and PMF using microscopic images from bone marrow smears, potentially complementing core biopsy analysis.

## Supplementary Information

Below is the link to the electronic supplementary material.

Supplementary Material 1

## Data Availability

No datasets were generated or analysed during the current study.
