# Supplementary material for: Deep learning differentiates myeloproliferative neoplasms and predicts JAK2 and CALR mutations from bone marrow smears
Source: Ann Hematol. 2026 Aug 25;105(9):393. doi: 10.1007/s00277-026-07257-w (PMC13518502; doi:10.1007/s00277-026-07257-w)
Supplement: Supplementary file 1 [file 277_2026_7257_MOESM1_ESM.pdf]

Supplemental Tables

Table S1. Performance of myeloproliferative neoplasm subtype prediction from bone marrow smears

	**Training Set (ET / PMF / PV)**	**Test Set (ET / PMF / PV)**	**Accuracy**	**Macro ROC-AUC**	**Macro F1 Score**	**Macro Precision**	**Macro Recall**
**Fold 1**	316 (119/124/73)	157 (58/62/37)	0.75	0.85	0.68	0.70	0.66
**Fold 2**	318 (118/126/74)	155 (59/60/36)	0.76	0.83	0.69	0.72	0.66
**Fold 3**	322 (120/128/74)	151 (57/58/36)	0.73	0.84	0.68	0.69	0.67
**Mean ± std**	-	-	0.75 ± 0.02	0.84 ± 0.01	0.68 ± 0.01	0.70 ± 0.02	0.66 ± 0.01

Abbreviations: essential thrombocythemia (ET), primary myelofibrosis (PMF), polycythemia vera (PV), receiver operating characteristic area under the curve (ROC-AUC), standard deviation (std)

Table S2. ***JAK2*** **mutation status prediction in essential thrombocythemia**

	Training Set (mut/N)	Test Set (mut/N)	Accuracy	Macro ROC-AUC	Macro F1 Score	Macro Precision	Macro Recall
**Fold 1**	81/119	37/56	0.68	0.74	0.63	0.64	0.63
**Fold 2**	81/119	37/56	0.62	0.73	0.61	0.63	0.65
**Fold 3**	78/116	40/59	0.68	0.71	0.60	0.62	0.60
**Mean ± Std**	-	-	0.66 ± 0.03	0.73 ± 0.01	0.61 ± 0.02	0.63 ± 0.01	0.63 ± 0.03

Abbreviations: number of patients bearing a mutation (mut) amongst all (N) patients in the set, receiver operating characteristic area under the curve (ROC-AUC), standard deviation (std)

Table S3. ***CALR*** **mutation status prediction in essential thrombocythemia**

	Training Set (mut/N)	Test Set (mut/N)	Accuracy	Macro ROC-AUC	Macro F1 Score	Macro Precision	Macro Recall
**Fold 1**	25/116	13/59	0.73	0.75	0.65	0.64	0.67
**Fold 2**	25/119	13/56	0.77	0.78	0.70	0.69	0.72
**Fold 3**	26/119	12/56	0.67	0.72	0.62	0.62	0.67
**Mean ± Std**	-	-	0.72 ± 0.05	0.75 ± 0.03	0.66 ± 0.04	0.65 ± 0.04	0.69 ± 0.03

Abbreviations: number of patients bearing a mutation (mut) amongst all (N) patients in the set, receiver operating characteristic area under the curve (ROC-AUC), standard deviation (std)

Table S4. ***JAK2*** **mutation status prediction in primary myelofibrosis**

	Training Set (mut/N)	Test Set (mut/N)	Accuracy	Macro ROC-AUC	Macro F1 Score	Macro Precision	Macro Recall
**Fold 1**	79/122	41/62	0.68	0.62	0.62	0.66	0.62
**Fold 2**	82/125	38/59	0.62	0.67	0.62	0.63	0.64
**Fold 3**	80/123	40/61	0.69	0.70	0.67	0.67	0.68
**Mean ± Std**	-	-	0.66 ± 0.04	0.67 ± 0.03	0.64 ± 0.03	0.65 ± 0.02	0.65 ± 0.03

Abbreviations: number of patients bearing a mutation (mut) amongst all (N) patients in the set, receiver operating characteristic area under the curve (ROC-AUC), standard deviation (std)

Table S5. ***CALR*** **mutation status prediction in primary myelofibrosis**

	Training Set (mut/N)	Test Set (mut/N)	Accuracy	Macro ROC-AUC	Macro F1 Score	Macro Precision	Macro Recall
**Fold 1**	32/121	17/63	0.71	0.73	0.67	0.67	0.71
**Fold 2**	34/124	15/60	0.66	0.69	0.62	0.62	0.65
**Fold 3**	33/125	16/59	0.62	0.68	0.61	0.65	0.68
**Mean ± Std**	-	-	0.66 ± 0.05	0.70 ± 0.02	0.63 ± 0.03	0.65 ± 0.03	0.68 ± 0.03

Abbreviations: number of patients bearing a mutation (mut) amongst all (N) patients in the set, receiver operating characteristic area under the curve (ROC-AUC), standard deviation (std)
